# Possible involvement of genome-wide near-haploidy and *MEN1* mutation in the molecular pathogenesis of pituitary carcinoma: Analysis by whole exome sequencing

**DOI:** 10.1093/noajnl/vdag146

**Published:** 2026-06-02

**Authors:** Satoru Kida, Takuma Nakashima, Satoshi Baba, Shinichiro Koizumi, Miho Yamashita, Sumihito Nobusawa, Chie Inomoto, Hiroyoshi Kino, Eiichi Ishikawa, Kazuhito Takeuchi, Tetsuya Nagatani, Ryuta Saito, Shingo Fujio, Ryosuke Hanaya, Joji Ishida, Shota Tanaka, Isao Date, Yasuyuki Kinoshita, Akira Taguchi, Yoshitaka Narita, Hiromichi Suzuki, Kazuhiko Kurozumi

**Affiliations:** Department of Neurosurgery, Hamamatsu University School of Medicine, Shizuoka, Japan; Division of Brain Tumor Translational Research, National Cancer Center Research Institute, Tokyo, Japan; Department of Pathology, Hamamatsu University School of Medicine, Shizuoka, Japan; Department of Neurosurgery, Hamamatsu University School of Medicine, Shizuoka, Japan; Department of Endocrinology and Metabolism, Hamamatsu University School of Medicine, Hamamatsu, Japan; Department of Human Pathology, Gunma University Graduate School of Medicine, Maebashi, Japan; Department of Pathology, Tokai University School of Medicine, Kanagawa, Japan; Department of Neurosurgery, Institute of Medicine, University of Tsukuba, Ibaraki, Japan; Department of Neurosurgery, Institute of Medicine, University of Tsukuba, Ibaraki, Japan; Department of Neurosurgery, Nagoya University Graduate School of Medicine, Aichi, Japan; Department of Neurosurgery, Japanese Red Cross Aichi Medical Center Nagoya Daini Hospital, Aichi, Japan; Department of Neurosurgery, Nagoya University Graduate School of Medicine, Aichi, Japan; Department of Neurosurgery, Graduate School of Medical and Dental Sciences, Kagoshima University, Kagoshima, Japan; Department of Neurosurgery, Graduate School of Medical and Dental Sciences, Kagoshima University, Kagoshima, Japan; Department of Neurological Surgery, Okayama University Graduate School of Medicine, Dentistry and Pharmaceutical Sciences, Okayama, Japan; Department of Neurological Surgery, Okayama University Graduate School of Medicine, Dentistry and Pharmaceutical Sciences, Okayama, Japan; Department of Neurological Surgery, Okayama University Graduate School of Medicine, Dentistry and Pharmaceutical Sciences, Okayama, Japan; Department of Neurosurgery, Graduate School of Biomedical and Health Sciences, Hiroshima University, Hiroshima, Japan; Department of Neurosurgery, Graduate School of Biomedical and Health Sciences, Hiroshima University, Hiroshima, Japan; Department of Neurosurgery and Neuro-Oncology, National Cancer Center Hospital, Tokyo, Japan; Division of Brain Tumor Translational Research, National Cancer Center Research Institute, Tokyo, Japan; Department of Neurosurgery, Hamamatsu University School of Medicine, Shizuoka, Japan

**Keywords:** *MEN1*, near-haploid, pituitary carcinoma, pituitary neuroendocrine tumor, whole exome sequencing

## Abstract

**Background:**

Pituitary carcinoma is a rare tumor, accounting for 0.12% of pituitary neuroendocrine tumors (PitNETs), that is derived from adenohypophyseal cells with craniospinal or systemic metastasis. While most PitNETs have a benign course, pituitary carcinoma has a poor prognosis, and its underlying molecular mechanism remains unclear. Here, we explored the molecular pathogenesis of pituitary carcinoma using whole exome sequencing (WXS).

**Methods:**

Tumor tissue samples were collected from patients diagnosed with pituitary carcinoma from April 2000 to March 2024 through multi-institutional joint research. DNA extraction and WXS were performed on the collected samples, and we analyzed the obtained data.

**Results:**

Five samples from four patients were collected (four men, mean age 57.5 years). DNA extraction and WXS were performed on the five samples. Gene mutation analysis revealed *MEN1* mutations in three samples. Copy number analysis showed unstable chromosomal conditions of genome-wide hypoploidy, which can be called near-haploidy, in three samples.

**Conclusions:**

This study showed that pituitary carcinoma had genome-wide hypoploidy, that is, genome-wide chromosomal instability and *MEN1* mutations as a possible recurrent mutation. Accumulated or original chromosomal instability may lead to the development of pituitary carcinoma from PitNET, and *MEN1* mutations may also play an important role in the pathogenesis of pituitary carcinoma. These findings may lead to strategies for earlier diagnosis and new treatments for this disease.

Key Points

- Pituitary carcinoma is a rare, aggressive malignancy with an unclear molecular basis.
- Exome sequencing revealed hypoploidy and *MEN1* mutations as recurrent events.
- Findings suggest a novel pathway of transformation from PitNETs to carcinoma.

Importance of the Study

Pituitary carcinoma is an exceptionally rare and aggressive tumor derived from adenohypophyseal cells, and its molecular pathogenesis remains poorly understood. In this multi-institutional study, we performed whole exome sequencing of pituitary carcinoma samples and identified genome-wide hypoploidy, reflecting chromosomal instability, along with *MEN1* mutations as possible recurrent events. To our knowledge, this represents the first report linking these genomic changes to pituitary carcinoma. These results provide novel insights into the mechanisms of malignant transformation in pituitary tumors and may contribute to the development of new diagnostic markers and therapeutic strategies.

Pituitary carcinoma is a rare pituitary tumor, accounting for 0.12% of all pituitary neuroendocrine tumors (PitNETs). It is strictly defined as “a tumor of adenohypophyseal cells that metastasizes craniospinally or systemically,” independent of histopathological findings.^1^ Most PitNETs follow a benign clinical course. However, PitNET can metastasize to develop pituitary carcinoma after an average of 5-6 years from initial diagnosis, with relatively large variability (in some cases, after months to decades).^2^^,^^3^ Once they metastasize and are diagnosed as pituitary carcinoma, the prognosis deteriorates drastically, with a median survival of less than 2-3 years.^2^^,^^3^ While the diagnosis of pituitary carcinoma and histology are independent, the lactotroph and corticotroph phenotypes account for the most frequently reported immunophenotype.^1^^,^^4^ Pituitary carcinoma also has a higher Ki-67 index of 11%-12% than that of PitNETs.^2^^,^^3^^,^^5^

Along with the nomenclature change of “pituitary adenoma” to “PitNET” in the World Health Organization (WHO) 2022 classification of pituitary tumors, the term “pituitary carcinoma” is also advocated to be changed to “metastatic PitNET.”^6^ As metastasis does not cause a malignant change in tumor histological findings, the term metastatic PitNET may more appropriately describe this tumor. Although extremely rare, very high-grade anterior pituitary tumors that should be termed neuroendocrine carcinoma have also been reported,^7-9^ resulting in difficulty for the clear-cut classification of this disease. Moreover, no conclusive biological and molecular markers specific to pituitary carcinoma have been reported. Furthermore, why only a few PitNETs metastasize and develop a malignant course, while most PitNETs have a benign course, remains to be clarified.

In the past two decades, technology for omics analysis has been advancing, and next-generation sequencing (NGS) has become widely available. Comprehensive analyses using NGS for various diseases, including neoplasms and genetic disorders, have been conducted, which have allowed elucidation of etiologies and treatment development. Here, we performed whole exome sequencing (WXS) analysis of pituitary carcinoma through multi-institutional joint research to explore the molecular mechanism of this tumor type.

## Methods

### Patients and Tumor Samples

Prior to the research, we applied to the Ethics Committee of Hamamatsu University School of Medicine for batch review of this study as multi-institutional joint research and obtained ethical approval (Study No. 22-197). Patients diagnosed with pituitary carcinoma with successful tumor sample collection from April 2000 to March 2024 at our institute and the joint research institute were included in this study. Tumor samples, including both primary and metastatic lesions, were obtained from patients during surgery. Formalin-fixed paraffin-embedded (FFPE) tumor sample blocks or sections attached to microscope slides were collected from each institute via refrigerated delivery.

### Sample Preparation

For FFPE blocks, 9-12 sections (10 µm thick) were made from the block. Excess paraffin was removed, and the tumor area was macrodissected. Next, 3-4 sections were placed in a 2 mL nucleic acid low-binding microtube.

For FFPE sections, 9-12 sections (10 µm thick) and a hematoxylin-eosin (HE)-stained specimen (or an unstained 4 µm section for HE staining) were prepared at each institute. After the sections were transported to our institute, the excess paraffin was removed, and the tumor area was macrodissected. Next, 3-4 sections were placed in a 2 mL nucleic acid low-binding microtube.

### DNA Extraction and WXS

After preparation, tumor samples were sent to Rhelixa, Inc. (Tokyo, Japan) for DNA extraction and WXS. The total DNA was extracted from tumor samples using a GeneRead DNA FFPE kit (QIAGEN, Hilden, Germany) following the manufacturer’s protocol. Quality checks of extracted DNA were performed using NanoDrop One (Thermo Fisher Scientific, Waltham, MA, USA) and TapeStation (Agilent Technologies, Santa Clara, CA, USA). WXS was performed using a SureSelect Human All Exon V6 (Agilent Technologies) on a NovaSeq 6000 (Illumina, San Diego, CA, USA).

### WXS Data Analysis

#### Sequencing alignment of WXS data

Sequencing reads were mapped to the 1000 Genomes Project GRCh38 human genome reference sequence (ftp://ftp.1000genomes.ebi.ac.uk/vol1/ftp/technical/reference/GRCh38_reference_genome/GRCh38_full_analysis_set_plus_decoy_hla.fa), using Burrows-Wheeler alignment—MEM version 0.7.17 with the “-T 0 -Y” parameter.^10^ Duplicates were marked using biobambam v.2.0.146, and base quality scores were recalibrated using GATK v.4.2.0.0.^11^

#### Somatic Variant Calling

Somatic mutations were identified using four variant callers: MuTect2,^12^ EBcall,^13^ Strelka2,^14^ and Varscan2^15^ for single nucleotide variants (SNVs) and five variant callers, including SvABA,^16^ for indels,^16^ as we previously described.^17^ In brief, MuTect2 was run following the best practices workflows. EBCall v.0.2.2, Strelka v.2.9.10, and SvABA v0.2.1 were run with the default setting. Varscan2 v.2.4.4 was run with parameters “--strand-filter 0--min-var-freq 0.08--p-value 0.10.” The results were filtered using the “fpfilter” function with the option “-dream3-settings.” Strelka and Varscan2 are unable to run tumor samples without normal controls, and therefore, we used an in-house mixed control BAM. For each caller, the variants listed in the in-house panel of normals were discarded. We used coding variants that were identified by at least two callers, with the requirement that at least one of them was MuTect2 or Strelka2.

#### CNV Calling

Copy number variants (CNVs) were analyzed following the GATK4 Somatic CNV discovery workflow with the advanced smoothing parameters “--kernel-variance-copy-ratio 2.0--kernel-scaling-allele-fraction 0.” We performed extensive smoothing to denoise the result as follows: segments too short (<1,000 bp), with too low seg value (<−10), or supported by a small number of probes (15 probes) were removed; copy neutral for the centromere and telomere regions was forced because these regions are difficult to call CNV accurately; and the minimum difference of absolute copy number between adjacent segments to the mean copy number was repeatedly flattened until the minimum difference among segments exceeded 0.2.

The denoised copy ratio represented a normalized and noise-corrected relative metric used to infer CNVs from sequencing data. Deviations from the copy ratio 1 indicate copy number alterations such as gain (ratio > 1) or loss (ratio < 1). B-allele frequency (BAF) represents the proportion of the minor allele (B allele) within a specific genomic region, which is used complementarily with the denoised copy ratio. On the basis of the fluorescence in situ hybridization (FISH) validation data, the denoised copy ratio was calibrated by setting regions with a copy number of two as the baseline (ratio = 1).

### FISH Analysis

To assess the absolute gene copy number in samples, FISH was performed. Dual-probe hybridization was conducted using 4-μm-thick FFPE tissue sections, as described previously.^18^ FISH probes were prepared from bacterial artificial chromosome clones RP11-59K17 (6q22.1), RP11-149I2 (9p21.3), RP11-281O15 (5q35.3), and RP11-715A21 (10q22.3); the former two and the latter two were labeled with ENZO Orange-dUTP and ENZO Green-dUTP (Abbott Molecular Inc., Des Plaines, IL, USA), respectively. Metaphase FISH to verify clone mapping positions was performed using the peripheral blood cell cultures of a healthy donor.

## Results

### Characteristics of the Patients and Tumor Samples

A total of five tumor samples obtained from four patients with pituitary carcinoma were collected. All four patients were male, with an average age of 57.5 years. The clinical characteristics, treatment history, and outcomes of patients with pituitary carcinoma are shown in Table 1. No patient had a diagnosis of multiple endocrine neoplasia type 1 (MEN1). The tumor subtypes included lactotroph tumor (n = 1), two somatotroph tumors (n = 2), and corticotroph tumor (n = 1). Metastatic sites varied and included the anterior skull base, nasal cavity, lymph node, liver, bone, and cerebrum. All four patients underwent chemotherapy with temozolomide; one patient later received carboplatin plus etoposide, with subsequent pembrolizumab. Radiation therapy was usually given as fractionated radiotherapy and/or stereotactic radiosurgery, targeting both primary and metastatic lesions as appropriate. The time from initial onset to diagnosis varied considerably, ranging from 0.5 to 12 years. Survival after diagnosis ranged from 21 to 60 months.

**Table 1.** Clinical characteristics, treatment history, and outcomes of patients with pituitary carcinoma

Patient	Age	Sex	MEN1	Tumor subtype	Metastatic site	Chemotherapy	Radiotherapy	Time from initial onset to diagnosis	Survival after diagnosis
A	63	M	No	Lactotroph, functioning	Anterior cranial base	TMZ	RT + SRS for PL	5 years	31 months
B	40	M	No	Somatotroph, non-functioning	Nasal cavity, cervical lymph node, liver, bone	TMZ	SRS for PL	4 years	24 months
C	69	M	No	Somatotroph, non-functioning	Thoracic vertebra	TMZ	SRS for PL, RT + SRS for ML	12 years	60 months
D	58	M	No	Corticotroph, non-functioning	Liver, bone, cerebrum	TMZ → CBDCA + VP-16 → Pembrolizumab	RT for PL + ML	0.5 years	21 months

Abbreviations: CBDCA, carboplatin; MEN1, multiple endocrine neoplasia type 1; ML, metastatic lesion; PL, primary lesion; RT, radiotherapy; SRS, stereotactic radiosurgery; TMZ, temozolomide; VP-16, etoposide.

The characteristics of the five tumor samples (A, B-1, B-2, C, and D) from the four patients (patients A-D) are shown in Table 2. Samples B-1 and B-2 were obtained from patient B and were from the local recurrent lesion and metastatic lesion, respectively. Samples A and C were from metastatic lesions, while sample D was from the primary lesion. Samples A and C were collected following chemoradiotherapy and chemotherapy, respectively, and the other samples were obtained before treatment.

**Table 2.** Characteristics of pituitary carcinoma tumor samples

Sample	Sample site	Hormone	Transcription factor	Initial/recurrence	Chemotherapy before sample collection	Radiotherapy before sample collection	p53	Ki-67
A	Anterior cranial base	PRL	PIT-1	Recurrence	TMZ	50 Gy/25 fr	NA	22%
B-1	Pituitary	None	PIT-1	Recurrence	None	None	Positive	10%
B-2	Nasal cavity	None	PIT-1	Recurrence	None	None	Positive	10%
C	Thoracic vertebra	GH	PIT-1	Recurrence	TMZ	None	Negative	10%
D	Pituitary	ACTH	T-PIT	Initial	None	None	Positive (70%)	>70%

Abbreviations: ACTH, adrenocorticotropic hormone; GH, growth hormone; NA, not assessed; PRL, prolactin; TMZ, temozolomide.

The immunohistochemistry results for hormones revealed the following positive expression: prolactin (sample A), growth hormone (sample C), and adrenocorticotropic hormone (sample D); samples from patient B were negative for hormone expression. Immunohistochemistry for transcription factors showed PIT-1 expression in samples A, B-1, B-2, and C, while sample D showed positive T-PIT expression. Although Sample D was a recurrent lesion, it was obtained during the initial surgical resection at that site. The Ki-67 labeling index ranged from 10% to over 70%. Sample D exhibited strong positive staining for p53 and Ki-67, with an expression level of both factors exceeding 70%.

### Summary of Recurrent Mutations and CNVs

**Figure 1.**
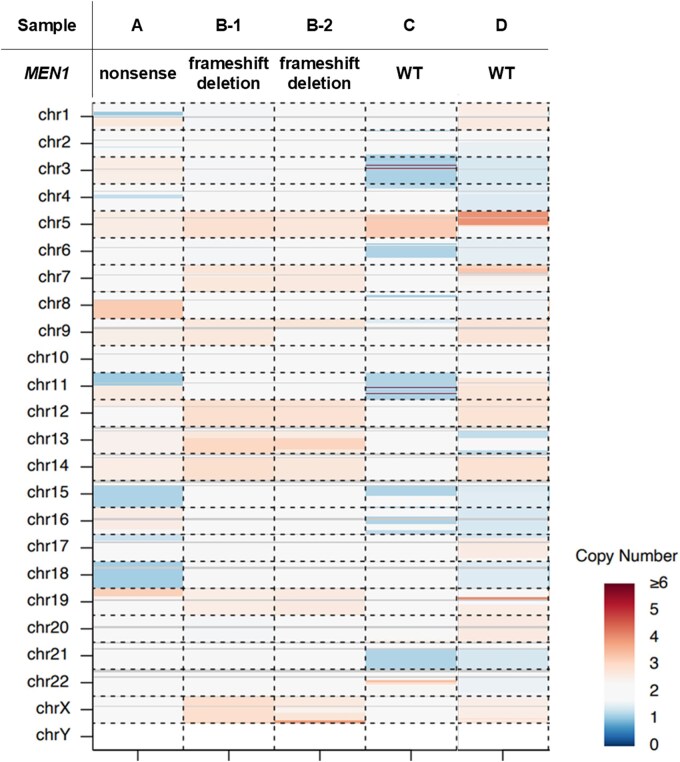
An overview of the recurrent truncated cancer gene mutations and copy number variations in the pituitary carcinoma samples. Of the genes known to be cancer drivers, only *MEN1* exhibited a recurrent truncated mutation in this study. Sample A harbored a *MEN1* nonsense mutation, and samples B-1 and B-2 exhibited frameshift deletions. A color-coded overview table of CNVs across the samples did not reveal any clear recurrent copy number alterations.

Figure 1 provides an overview of the recurrent mutations and CNVs in the pituitary carcinoma samples. As normal tissue controls were unavailable except for patient D, only truncated mutations observed in two or more cases were extracted from the variant called dataset. *DYNC1I2*, *SPTAN1*, *DST*, and *MEN1* mutations were recurrently identified in three samples. *PRUNE2*, *DNAJC11*, *UBE4B*, *LYST*, *TRA2B*, *KIAA0319*, *CNGB3*, *MPDZ*, *ABCC4*, and *MYT1* mutations were common among the two samples, excluding those exclusively shared between B-1 and B-2. These genes were examined for inclusion in the OncoKB Cancer Gene List, and *MEN1* was the only gene identified in this list. For *MEN1*, sample A harbored a nonsense mutation, and samples B-1 and B-2 exhibited frameshift deletions; samples C and D retained the wild-type sequence. We also found that, although not recurrent, sample D harbored a *TP53* nonsynonymous SNV (p.V172F) and *RB1* frameshift deletion (p.N290Mfs*11). However, these mutations were not detected in the other samples. No other alterations in cell cycle-related genes were detected in the focal CNV analysis.

The color-coded overview table of CNVs across the samples did not reveal any clear recurrent copy number alterations. However, widespread copy number alterations were present, suggesting unstable genomic states.

### Copy Number Analysis

**Figure 2.**
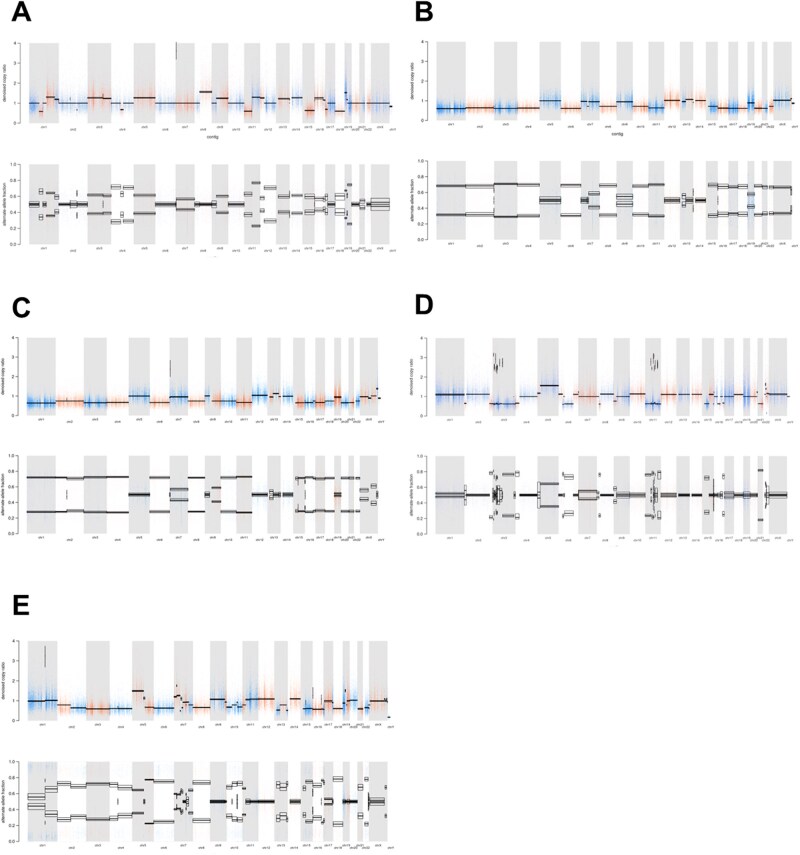
Copy number and B-allele frequency (BAF) profiles of pituitary carcinoma samples. (A) Sample A; (B) Sample B-1; (C) Sample B-2; (D) Sample C; (E) Sample D. The top panel shows the denoised copy ratio, and the bottom panel shows BAF. Copy ratio >1 indicates gain, and a copy ratio <1 indicates loss. (A, D) In samples A and C, the baseline of the copy ratio remains close to 1; on chromosomes of copy ratio 1, BAF clusters ≈0.5, indicating a diploid state. (B, C, E) In samples B-1, B-2, and D, the baseline of copy ratio was less than 1, and BAF distributions split toward ∼0.33 and ∼0.67, consistent with genome-wide hypoploidy and imbalanced genomic states markedly divergent from a normal diploid configuration.

We reviewed the copy number profiles of each sample, and three of the five samples exhibited genome‐wide hypoploidy (Figure 2). In sample A (Figure 2A), the baseline denoised copy ratio was approximately 1, and the BAF on those chromosomes clustered around 0.5, indicating a diploid state. Sample C (Figure 2D) exhibited an analogous pattern, also consistent with a diploid state. In stark contrast, samples B-1 (Figure 2B) and B-2 (Figure 2C) showed BAF values split at the 2:1 position rather than at 0.5 across many chromosomes, with the denoised copy ratio baseline markedly below 1, suggesting genome-wide hypoploidy. While sample D (Figure 2E) displayed even more uneven copy number alterations, the baseline denoised copy ratio lay substantially below 1, and the BAF also clustered at a 2:1 ratio, similar to samples B-1 (Figure 2B) and B-2 (Figure 2C), indicating genome-wide hypoploidy and imbalanced genomic states markedly divergent from a normal diploid configuration.

### FISH Analysis

From the copy number analysis, genome-wide copy number alterations suggestive of ploidy abnormalities were observed in samples B-1, B-2, and D. Therefore, we performed FISH to validate the chromosomal copy number. Probes targeting 5q35.3 (green) and 6q22.1 (red), along with 9p21.3 (red) and 10q22.3 (green), were used, and the FISH signal counts were compared with the denoised copy ratio values of the matched area to assess chromosomal copy numbers. In samples B-1 and D, only a single FISH signal per probe was observed at the baseline denoised copy ratios, indicating that the tumor’s baseline chromosome was present in one copy, demonstrating genome-wide hypoploidy that can be characterized as near-haploid. Sample B-2 exhibited cell enlargement, and many cells displayed exactly twice the number of signals compared with that in B-1, suggesting that a whole-genome doubling event occurred from a near-haploid state. In samples A and C, the comparison between the denoised copy ratios and FISH signal counts indicated that the cells were typically diploid.

**Figure 3.**
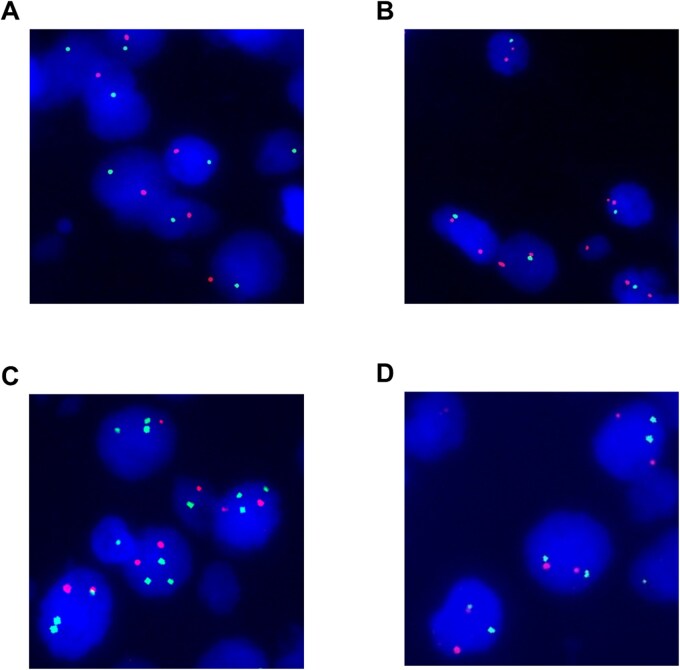
Representative images of fluorescence in situ hybridization (FISH) of tumor samples. FISH analysis of sample D using (A) 5q35.3 (green) and 6q22.1 (red) probes and (B) 9p21.3 (red) and 10q22.3 (green) probes. FISH analysis of sample A using (C) 5q35.3 (green) and 6q22.1 (red) probes and (D) 9p21.3 (red) and 10q22.3 (green) probes. In (A) and (B), the signals, which should normally be observed as two copies in diploid cells, are reduced to approximately half.

Representative images are shown in Figure 3. For sample D (Figure 3A and B), the 5q35.3 (green) and 6q22.1 (red) probes (Figure 3A) and the 9p21.3 (red) and 10q22.3 (green) probes (Figure 3B) yielded only 1, 1, 2, and 1 signals, respectively, confirming a near-haploid karyotype. By contrast, sample A (Figure 3C and D) displayed 3, 2, 2, and 2 signals for the same probes, in agreement with a diploid chromosomal complement.

### Representative Case

**Figure 4.**
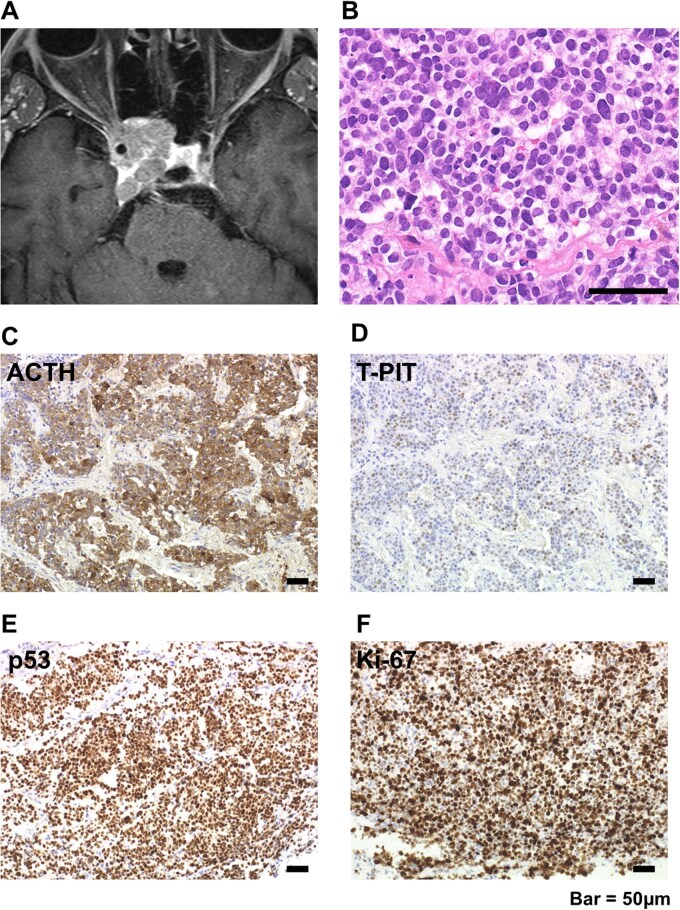
Imaging and findings of a 58-year-old male patient. (A) Contrast-enhanced T1-weighted image at the time of admission showing a multilobular, homogeneously enhanced pituitary tumor invading the right cavernous sinus. (B) High-power field image of hematoxylin-eosin staining of the tumor tissue. Tumor cells show a high nuclear/cytoplasmic ratio and proliferate in a solid growth pattern. Several mitoses can be seen. (C) Immunohistochemistry of ACTH in the tumor sample. (D) Immunohistochemistry for T-PIT in the tumor sample. (E) Immunohistochemistry of p53 in the tumor sample. (F) Immunohistochemistry for Ki-67 in the tumor sample.

A 58-year-old man was referred to our hospital for right oculomotor and abducens nerve palsy because of a rapidly growing pituitary tumor. He had a history of pituitary apoplexy two years earlier. Although the operation was planned, treatment of an abdominal aortic aneurysm incidentally discovered in the perioperative period was given priority, resulting in conservative follow-up for the pituitary apoplexy. At the time of admission to our hospital, magnetic resonance imaging showed an irregularly shaped, enlarging pituitary tumor invading the right cavernous sinus (Figure 4A). Endocrinologic examination suggested a nonfunctioning tumor. The patient underwent endoscopic transsphenoidal surgery, and intraoperative findings showed a solid fibrous tumor, which was quite different from the usual PitNET. Histological findings revealed that, in many areas, tumor cells with a high nuclear/cytoplasmic ratio and prominent nuclear atypia proliferated with solid and honeycomb-like growth, accompanied by numerous mitoses and necroses (Figure 4B). These findings clearly suggested carcinoma. In immunohistochemistry, ACTH and T-PIT were positive, indicating that the tumor cells were derived from corticotroph cells (Figure 4C and D). p53 was diffusely positive, and Ki-67 was positive in more than 70% of cells, suggesting that the tumor had very high proliferative potential (Figure 4E and F). On the basis of these results, this tumor was diagnosed as a neuroendocrine carcinoma developed from pituitary corticotroph cells.

After surgery, the patient was systemically examined for metastatic lesions, and no obvious metastatic lesions were found. He underwent extended focal irradiation (50 Gy/25 fr) to the tumor, and the primary lesion showed no recurrence on magnetic resonance images. However, liver and bone metastases had developed 6 months after surgery. Therefore, the patient was diagnosed with pituitary carcinoma. He was treated first with temozolomide, followed by carboplatin and etoposide. Cancer gene panel testing revealed a high tumor mutational burden. After failure of the above chemotherapy, he was treated with pembrolizumab, an immune checkpoint inhibitor. Pembrolizumab was effective for several months; however, he subsequently developed a distant metastatic brain tumor in the left occipital lobe and died from intratumoral hemorrhage 27 months after surgery.

## Discussion

### Summary of Results

In this study, we performed WXS on five tumor samples from four pituitary carcinoma patients. We found that genome-wide hypoploidy, which can be called near-haploidy, was present in three samples, and *MEN1* mutations were present in three samples. These results suggest that this kind of hypoploidy, as well as *MEN1* mutations, may be involved in the molecular pathogenesis of pituitary carcinoma.

### Near-Haploidy

The results of copy number analysis indicated that genome-wide hypoploidy, considered near-haploidy, may be involved in the development of pituitary carcinoma. Ploidy refers to the number of complete sets of chromosomes in a cell. Humans are diploid organisms, and thus each somatic cell has 22 pairs of autosomes and 2 sex chromosomes.^19^ Polyploidy means that the whole set of chromosomes in each somatic cell duplicates as three (triploidy), four, or more sets.^20^ Haploidy describes the state in which a cell contains a single complete set of chromosomes. In diploid organisms, while somatic cells are normally diploid (2n), haploid (n) cells carry only one set of chromosomes. In multicellular organisms, the haploid state is restricted to gametes—spermatozoa and ova—which deliver half the genetic complement to the next generation.^21^

The association between tumors and aneuploidy, the reduplication or loss of some sets of chromosomes, has been controversial for more than a century.^22^ To date, only a few studies have investigated the association between PitNET and ploidy. These reports indicate that aneuploidy is more common in functional PitNETs than in nonfunctional ones.^23^^,^^24^ However, these studies did not address the impact of ploidy level on PitNET malignancy. To the best of our knowledge, no studies have reported the involvement of genome-wide hypoploidy indicative of an essentially near-haploid state in pituitary carcinoma.

Regarding the relationship between aneuploidy and various other malignant tumors, hyperploidy is reported far more often, while hypoploidy is less common.^25^^,^^26^ Although less common, hypoploidy has been associated with poor prognosis in certain malignant tumors. Large studies in breast cancer showed that hypoploid tumors with a DNA index below 0.95 have an extremely poor prognosis and are an independent poor prognostic factor over other clinicopathologic factors.^27^ Genomic analysis of hypodiploid acute lymphoblastic leukemia revealed the existence of two subtypes, near-haploid and low-hypodiploid, each with abnormalities in the Ras/PI3K pathway and *TP53*, *RB1*, and *IKAROS* family genes.^28^ Studies in mesothelioma have shown that inactivation of *SETDB1* and aberrant expression of *TP53* in the near-haploid form are deeply involved in the tumor suppression mechanism.^29^ One hypothesis we propose for pituitary carcinoma genesis is that genome-wide hypoploidy precipitates extensive loss of heterozygosity across multiple chromosomes,^30^ thereby unmasking and concentrating oncogenic alleles. In this setting, a single activated oncogene can act as the sole driver of malignant neoplastic transformation in PitNET cells. Such hypoploidy-induced loss of heterozygosity may thus create a genomic landscape in which monoallelic oncogene dependence becomes the critical event that initiates and sustains pituitary carcinoma. PitNETs with near-haploid tumor cells may thus develop pituitary carcinoma as a result of genome-wide chromosomal instability.

### MEN1 Mutations

In this study, gene mutation analysis suggested that *MEN1* mutations may also be associated with the pathogenesis of pituitary carcinoma. The *MEN1* gene is located on chromosome 11q13 and encodes the menin protein. *MEN1* gene mutations cause MEN1, an autosomal dominant tumor syndrome characterized by the occurrence of two or more endocrine tumors, represented by parathyroid tumors, pancreatic islet tumors, and PitNETs.^31^ In patients with MEN1, 40% develop PitNETs; however, less than 3% of all PitNET patients have MEN1^31^; in other words, most PitNET patients are non-MEN1. In a MEN1 multicenter study conducted in France and Belgium, MEN1 PitNETs were more likely to exhibit macrotumors larger than 1 cm in diameter compared with non-MEN1 PitNETs (85% vs. 42%, *P* < .001).^32^ From this viewpoint, PitNETs associated with MEN1 may be somewhat aggressive. In contrast, studies including many MEN1 patients reported that MEN1 did not affect the malignant transformation of PitNETs, including the development of pituitary carcinoma.^32^^,^^33^ In fact, only a few cases of pituitary carcinoma in MEN1 patients were reported.^34^^,^^35^ These results indicate that MEN1 may not directly cause the development of pituitary carcinoma from PitNET. However, given that pituitary carcinoma is a rare tumor, accounting for only 0.12% of all PitNETs, even though most MEN1 patients do not develop pituitary carcinoma, *MEN1* mutations may play some important role in the molecular mechanism to develop pituitary carcinoma among the patients with this tumor.

Menin, originally identified as a tumor suppressor, has recently been shown to have a pro-oncogenic function.^36^ In the present study, two of the four patients had *MEN1* mutations, while none of the patients had a history of MEN1. Given that the *MEN1* gene has a pro-oncogenic function, certain somatic mutations in *MEN1* gene distinct from germline mutations may underlie the tumorigenesis of pituitary carcinoma. While the current study included a small sample size, the fact that two of the four patients had the same *MEN1* mutation may suggest that *MEN1* mutations may be recurrent cancer driver mutations that are important in the etiology of the disease.

### Comparison With Previously Reported Gene Mutations

We also surveyed the literature for genes previously implicated in pituitary carcinoma and examined our WXS data to determine whether those same mutations were present in our study data. In the WHO 2022 classification, TP53 immunohistochemistry was removed as a criterion suggestive of aggressiveness in PitNETs. Nevertheless, *TP53* mutations are still regarded as molecular alterations with a potential negative prognostic impact in PitNETs.^37^ In our cohort, *TP53* mutation was identified in only sample D. Recent studies have reported that *TP53* mutations occur predominantly in aggressive corticotroph PitNETs^37-39^; however, such mutations are comparatively uncommon in other subtypes. Sample D was a silent corticotroph tumor belonging to the T-PIT lineage, which is consistent with these studies.

In a prior study of alpha thalassemia/mental retardation syndrome X-linked (*ATRX*) gene alterations in corticotroph aggressive PitNETs/pituitary carcinomas, loss-of-function *ATRX* mutations were detected in 5 of 18 cases (28%), with an especially high frequency in the corticotroph subtype (4 of 12 cases, 33%).^38^ Panel sequencing of nine *ATRX*-negative tumors from the cohort uncovered a variety of loss-of-function alterations, including nonsense mutations, frameshift insertions/deletions, and large intragenic deletions. While studies focusing specifically on aggressive PitNETs and pituitary carcinomas remain limited, a recent systematic review of *ATRX* expression in PitNETs reported *ATRX* loss in only 20 of 513 cases (3.9%).^40^ Notably, 12 of these 20 *ATRX*‑negative tumors (60%) were corticotroph PitNETs. Furthermore, of the seven pituitary carcinomas with *ATRX* loss included in the review, six were of the corticotroph subtype. In the present study, *ATRX* alterations were identified in only one sample (sample A), which harbored a nonsynonymous SNV. Four of the five tumors analyzed in this study were of the PIT-1 lineage, and thus, the paucity of *ATRX* mutations may reflect this lineage bias. The previous report also noted coexisting *TP53* mutations in two of the five *ATRX*-negative pituitary carcinomas; in our series, *TP53* alterations were likewise rare, with only sample D exhibiting a nonsynonymous SNV.

While only a few cases have been reported, two pituitary carcinoma patients have been shown to harbor both *LAG3* overexpression and *DICER1* mutation.^41^ In our series, *LAG3* nonsense mutations were identified in samples B-1 and B-2 (both from patient B), whereas nonsynonymous SNVs in *DICER1* were detected in samples A, B-1, and D. *DICER1* is regarded as a tumor suppressor gene and the causative gene for the hereditary tumor predisposition syndrome known as DICER1 syndrome. The development of DICER1 syndrome is thought to occur through a two-hit mechanism, in which a germline mutation in one allele is followed by a somatic mutation in the second allele.^42^ None of the patients in this study were suggestive of DICER1 syndrome, and all three *DICER1* variants were nonsynonymous SNVs rather than truncated mutations. Nevertheless, given the near-haploid genomic context described above, “a single-hit” in *DICER1* may contribute to driving tumorigenesis for pituitary carcinoma.

### Limitations

This study has several limitations. First, the number of samples was small, and future studies with a larger sample size are required to strengthen the findings. Second, owing to the absence of matched normal tissues, such as blood samples, except for one sample, it was not possible to determine whether the detected SNVs represent germline single-nucleotide polymorphisms or tumor-specific point mutations. Moreover, sequence data alone cannot reliably distinguish between germline and somatic variants. Finally, additional multi‑omics approaches—including genomics, transcriptomics, proteomics, and epigenomic analyses—might have helped achieve a more comprehensive understanding of the genetic alterations in pituitary carcinoma. In the future, if multi‑omics analyses identify potential therapeutic targets, in vivo studies using animal models such as *MEN1* knockout mice may provide a valuable platform for exploring novel treatment strategies for pituitary carcinoma.

## Conclusion

Our results indicate that PitNETs with global hypoploidy may develop pituitary carcinoma as a result of accumulated or original genome-wide chromosomal instability, and *MEN1* mutations may be a key genetic alteration involved in the pathogenesis of pituitary carcinoma. The potential involvement of genome-wide hypoploidy and *MEN1* mutations in the molecular pathogenesis of pituitary carcinoma has important implications for further research. These findings may lead to a better understanding of the molecular pathogenesis of pituitary carcinoma and contribute to earlier diagnosis and new treatments.

## Data Availability

The data supporting the findings of this study are currently under preparation for deposition in a public repository and will be made publicly available once deposition has been completed.
